# How is physicians’ implicit prejudice against the obese and mentally ill moderated by specialty and experience?

**DOI:** 10.1186/s12910-022-00815-7

**Published:** 2022-08-24

**Authors:** Chloë FitzGerald, Christian Mumenthaler, Delphine Berner, Mélinée Schindler, Tobias Brosch, Samia Hurst

**Affiliations:** 1 iEH2 (Institute for Ethics, History and the Humanities), Faculty of Medicine, University of Geneva, Geneva, Switzerland; 2 Department of Information Science, Geneva School of Business Administration, Geneva, Switzerland; 3 University of Applied Sciences and Arts, Western Switzerland, Delémont, Switzerland; 4 CISA (Swiss National Centre for Affective Sciences), University of Geneva, Geneva, Switzerland; 5 Department of Psychology, University of Geneva, Geneva, Switzerland

**Keywords:** Implicit bias, Prejudice, Stereotype, Specialization, Experience, Training, Medical education, Doctor–patient relationships, Vulnerable populations, Mental health, Obesity

## Abstract

**Background:**

Implicit prejudice can lead to disparities in treatment. The effects of specialty and experience on implicit obesity and mental illness prejudice had not been explored. The main objective was to examine how specializing in psychiatry/general medicine and years of experience moderated implicit obesity and mental illness prejudice among Swiss physicians. Secondary outcomes included examining the malleability of implicit bias via two video interventions and a condition of cognitive load, correlations of implicit bias with responses to a clinical vignette, and correlations with explicit prejudice.

**Methods:**

In stage 1, participants completed an online questionnaire including a clinical vignette. In stage 2, implicit prejudice pre- and post- intervention was tested using a 4 × 4 between-subject design including a control group. In stage 3, explicit prejudice was tested with feeling thermometers and participants were debriefed. Participants were 133 psychiatrists and internists working in Geneva, hospital-based and private practice. Implicit prejudice was assessed using a Weight IAT (Implicit Association Test) and a Mental Illness IAT. Explicit feelings towards the obese and the mentally ill were measured using Feeling Thermometers. A clinical vignette assessed the level of concern felt for a fictional patient under four conditions: control, obese, depression, obese and depression. Linear regression was conducted to test for association of gender, experience, and specialty with responses to vignettes, pre-intervention IATs and explicit attitudes, and to test for association of interventions (or control) with post-intervention IATs and explicit attitudes. Reported effect sizes were computed using Cohen’s d. Two-tailed *p* < 0.05 was selected as the significance threshold.

**Results:**

Compared to internists, psychiatrists showed significantly less implicit bias against mentally vs. physically ill people than internists and warmer explicit feelings towards the mentally ill. More experienced physicians displayed warmer explicit feelings towards the mentally ill and a greater level of concern for the fictional patients in the vignette than the less experienced, except when the patient was described as obese.

**Conclusions:**

Specialty moderates both implicit and explicit mental illness prejudice. Experience moderates explicit mental illness bias and concern for patients. The effect of specialty on implicit prejudice seems to be based principally on self-selection.

**Supplementary Information:**

The online version contains supplementary material available at 10.1186/s12910-022-00815-7.

## Background

Equal treatment of patients regardless of skin colour, weight, or other characteristics is part of the standard of care in medicine and physicians often cite helping others as a reason for practising medicine [1–6]. This standard of care has become even more relevant globally in the recent pandemic, where resources have often been scarce, and where disparities in death rates from Covid-19 correlated with race have been documented in the UK and the US [7, 8]. Fears among patients about discrimination due to factors such as obesity have also increased [9].

Implicit biases against stigmatised groups represent a threat to this standard of care. Implicit biases are associations, which have been characterised as automatic, uncontrollable, unconscious or arational [10], between a category attribute, e.g. being overweight, and a negative evaluation (implicit prejudice), or another category attribute, e.g. being lazy (implicit stereotype). In contrast to explicit biases, evaluations that a person makes consciously, implicit biases are typically manifest in non-verbal behaviour, such as the frequency of eye contact and physical proximity [11]. Their most disturbing aspect is the potential dissociation between what a physician explicitly intends (e.g. treat everyone equally) and the hidden influence of implicit associations on her decision-making and action (e.g. perceiving a black child’s pain as less severe than a white child’s and thus deciding not to prescribe the patient a pain medication) [12].

The effects of medical specialty and experience on implicit obesity and mental illness prejudice have not been explored in the literature. The principal objective of the study was to examine how specializing in psychiatry/general medicine and years of experience moderated implicit obesity and mental illness prejudice among Swiss physicians. The relationship between levels of implicit bias and clinical behaviour remains unclear [14] and we therefore evaluated physicians’ intended behaviour through their responses to a clinical vignette. Secondary outcomes included examining the malleability of implicit bias via two video interventions, a condition of cognitive load and correlations with explicit prejudice.

Prejudicial and stereotypical implicit attitudes are widespread among the worldwide population and can influence behaviour outside the laboratory [14, 15]. Many implicit biases are present even among the stigmatized outgroup, e.g. most obese people display an implicit prejudice against the obese [16]. Implicit biases among health care professionals and the potential influence on clinical care is a concern [17–19]. While they are not the whole picture, implicit biases are likely to play a part in health care disparities. In the US, racial health care disparities are widely documented and implicit racial bias is a possible contributing factor [20]. In the UK, a recent study found that a woman is five times more likely to die in childbirth or post-partum if she is black than if she is white [21]. Other health care disparities related to factors such as socio-economic status, sexual orientation and gender are possibly partly due to implicit biases [22].

Implicit bias training is widely on offer in the English-speaking world, despite a limited evidence base for effective interventions [23]. But what is the role of training and experience in medicine itself? Do more experienced physicians exhibit more or less implicit bias than trainees? Medical training might reasonably be expected to reduce bias with its emphasis on care and the impartial standard. One study found that mental health training correlated with more positive implicit and explicit attitudes to the mentally ill [24]. Experience in the field could potentially reduce prejudice by making physicians more sympathetic to patients. As they gain experience and knowledge, medical professionals may experience less cognitive strain and thus have more mental resources to devote to ensuring equal treatment of patients. It is possible that exposure to counterstereotipic exemplars that contradict an implicit stereotype can lessen it [25].

On the other hand, some experience will confirm stereotypes rather than contradict them, particularly given confirmation bias [26] and the important role of medical superiors, who may be biased, in training. Current evidence shows that contact with the stigmatised group sometimes decreases prejudice, but can also increase it, depending on the nature of the contact [27–30]. In addition, medical training encourages fast-thinking and shortcuts, which can increase incidence of implicit bias [31]. With experience and training a physician may become more confident about their ability to be impartial and thus more susceptible to bias. There is evidence that if we are confident about our moral capabilities we are more likely to display implicit biases because we register the goal of being impartial as being ‘achieved’ and monitor ourselves less [32]. There has also been research showing that clinical exposure can reduce empathy [33, 34].

In addition to training and experience, medical specialty may influence levels of implicit bias. One study found that paediatricians displayed less implicit race bias than other physicians [35]. The aforementioned study showing an effect of mental health training on bias towards the mentally ill could be a sign that choice of specialty and the subsequent training and environment influences bias. Specialty, training and experience are likely to interact in complex ways to produce potential effects on implicit bias. No studies have specifically explored the interaction of specialty and training/experience with implicit biases. This study aimed to fill this gap.

Implicit obesity prejudice and implicit mental illness prejudice were chosen partly because they are both tied to characteristics of patients that can be relevant for medical reasons. The former has been found to be present in health care professionals to a similar extent as in the general population [36]; there is less data on implicit mental health bias, but evidence indicates its presence in both physicians and the general population [24, 37–39]. The evidence indicating that mental health training reduced bias made this bias an obvious choice to explore. While black/white race bias has received the most attention due to its relevance in the US, it is not necessarily the most relevant to Swiss physicians, who may discriminate more based on country of origin rather than skin colour [40]. In addition, Geneva city and hospital are highly multicultural, meaning both patients and physicians in the hospital come from a multitude of different countries and typically speak several languages. Unlike race bias, obesity and mental illness bias have been found to exist as explicit biases in addition to implicit among health care professionals [13, 38, 39]. One explanation is that because obesity and mental health are medically relevant characteristics they fail to flag warning signs for prejudice with physicians, as, typically, race would.

Given findings of the presence of implicit obesity bias and implicit mental illness bias among physicians from many other countries [13], some level of implicit bias was expected to be found among Swiss physicians. On the other hand, culturally specific contexts could mean that within Swiss physicians, or specifically physicians in Geneva, we would find higher or lower levels than had been found in other studies [41].

It was hypothesized that training would have an effect on implicit biases, while the direction that this effect would take was uncertain given the mixed evidence to date. It was hypothesized that specializing in psychiatry would correlate with lower mental illness bias based on the one study showing an influence of mental health training on bias [35].

## Methods

### Study participants

Participants were recruited from psychiatry and general internal medicine. As mentioned in the Introduction, the evidence so far on the effects of experience and contact on prejudice is mixed. The specialties of psychiatry and general medicine were chosen because in examining bias against illness of the mind and a characteristic of the physical body (obesity) it was deemed interesting to compare specialists in the mind (psychiatrists) and those who did not specialise in the mind and thus could be expected to focus more on physical characteristics (general internists). Female physicians have been found to display weaker implicit bias against the obese than male physicians, hence gender differences in obesity bias were expected [36]. Participants were recruited from the Geneva University Hospitals and from private practices in Geneva by email or physical mail.

### Data collection

**Fig. 1:**
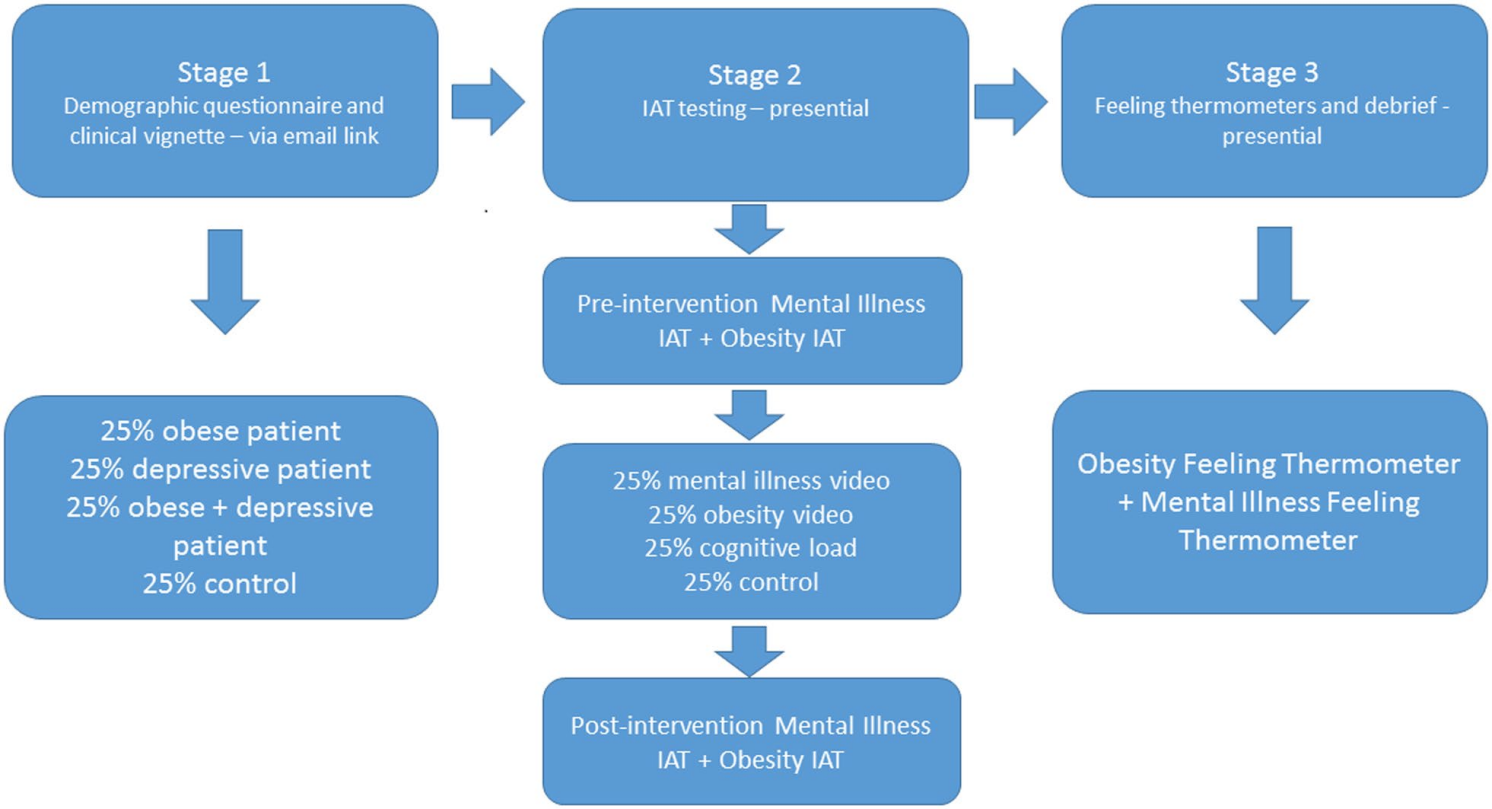
Experimental design

All participants provided written informed consent. The experiment was described as an investigation into implicit attitudes using a categorisation task with the aim of improving standards of clinical care. The IAT was not named nor were the words ‘bias’ and ‘prejudice’ mentioned to avoid influence on responses to the clinical vignette. The order of measures (Fig. 1) was chosen to mask the characteristics under study for as long as possible. The ethics committee approved this procedure for obtaining consent. Participants first completed a demographic questionnaire and a clinical vignette online. Patient characteristics were randomized in the vignette to create four groups each receiving a different version.

A face-to-face meeting was then held with each participant, who completed a Mental Illness IAT and a Weight IAT, followed by either one of two interventions, instructions for the cognitive load condition, or a control condition, then a repeat of both IATs, and finally each responded to two Feeling Thermometers to rate their feelings towards the obese and the mentally ill. The interviewer asked participants to refrain from discussing the contents of the study with colleagues. Interviews were conducted in the local language of the participants, French, and all materials and tests were in French. All methods were carried out in accordance with relevant guidelines and regulations.

### Measures

#### IATs

The most prevalent measure of implicit biases is the Implicit Association Test (IAT), a computerized task where participants rapidly categorize negatively and positively valenced words with images or words. The relative speed of association of, e.g. in a Race IAT, black faces with positively-valenced words (as compared to the other possible associations), indicates the level of bias [42].

Mental illness stimuli were taken from a previously tested IAT, comparing words for physical illnesses and words for mental illnesses paired with negatively and positively valenced words [39]. The Weight IAT used stimuli from the Project Implicit website: silhouette images of thin and obese people paired with negatively and positively valenced words [43]. Words were translated from English to French. D-score interpretation used by Project Implicit indicate a score greater than 0.15 as a slight bias, greater than 0.35 as a moderate bias, and greater than 0.65 as a strong bias. Negative scores represent the inverse association, namely an association between either mental health or obesity (as compared to physical health or thinness) with positive rather than negative words [42, 43].

#### Vignette

The vignette was taken from a study that found differences in clinical responses to pain correlated with patient gender [44]. It was translated from Portuguese to French and modified to create four versions: a control version with no medical history, a version where the BMI of the patient was 32 and thus indicated clinical obesity, a version were the patient had experienced depressive episodes, and a version were the patient had a BMI of 32 and had experienced depressive episodes. There were six questions that asked participants to evaluate pain intensity, clinical severity, clinical urgency and pain credibility on a scale of 1–7 (Additional file 1: S1 Appendix).

#### Feeling thermometer

These consisted in a continuous unnumbered line with ‘warm feelings’ written on the left side of the line and ‘cold feelings’ on the right. The range was 0–12, but the increments were not indicated on the actual line where participants marked a cross. Instead, the segments were designated as follows:

- 0–2.75 warm feelings
- 2.75–5.5 slightly warm feelings
- 5.5–6.5 neutral feelings
- 6.5–9.25 slightly cold feelings
- 9.25–12 cold feelings

Participants marked the point that represented their feelings towards the obese and the mentally ill. Lower scores represent warmer feelings. The feeling thermometer measures explicit feelings, thus is a measure of explicit bias.

### Interventions and cognitive load

Few interventions had been tested specifically on implicit weight bias and implicit mental illness bias and even fewer have produced significant results [45–50]. One of the methods that had been found to potentially reduce anti-age, racial and other implicit biases was perspective-taking, thought to increase empathy with the target group [51, 52]. We decided to test a version of perspective-taking, given that there was as much evidence that it worked as there was for other strategies and it was the one that most appealed to our research team. The participants were required to watch the short (1.25 min) extracts of videos made by the National Health Service in the UK before immediately retaking the IAT. The videos had been designed for training, to help physicians identify and empathise with their mentally ill and obese patients. They showed two genuine female patients in the UK talking about their clinical experiences with obesity and depression. The videos were subtitled in French and are available from the following website: https://www.unige.ch/medecine/ieh2/fr/recherche/groupe-samia-hurst-manjo/.

Given concerns regarding the test–retest reliability of the IAT [41, 53] and the possibility of a learning effect when participants completed the second IAT, we included a control group. In the control group, participants counted backwards in twos out loud in place of the intervention for the same length of time and then retook the IATs.

A fourth group, in place of the intervention, was instructed on how to proceed with the second set of IATs under a cognitive load condition, consisting in counting backwards in twos out loud during the intervention. They then proceeded to retake the IATs. The effects of implicit bias are thought to increase under stress and time pressure, common working conditions for physicians that can be simulated—albeit approximately—with a cognitive load. While previous research has shown that conditions of cognitive load increase implicit biases [54, 55], we were not aware of other research that looked directly at the effects of cognitive load while performing an IAT.

It was hypothesized that the video interventions would correlate with reduced levels of implicit prejudice and that the cognitive load condition would correlate with increased levels of implicit prejudice when compared with the control group.

### Statistical analysis

The sample size was computed using the software GPower and was based on an initial a priori power analysis for ANOVA, targeting a medium-sized interaction effect between two factors (specialty and experience) with two level each with an alpha of 0.05 and a power of 0.80, requiring a total sample size of N = 158 (40 per cell). Data was entered into the SPSS statistical software package for analysis. Participants’ socio-demographic characteristics were analysed using descriptive statistics. Inter item reliability (the questionnaire’s internal consistency) was tested using Cronbach’s alpha. We changed our analysis to enable treatment of IAT scores as continuous variables in response to reviewer comments. Linear regression was conducted to test for association of gender, experience, and specialty with responses to vignettes, pre-intervention IATs and explicit attitudes. Linear regression was also conducted to test for association of interventions (or control) with post-intervention IATs and explicit attitudes. To take possible confounding variables for this second analysis into account we also included gender, experience, and specialty. Reported effect sizes were computed using Cohen’s d. Two-tailed p < 0.05 was selected as the significance threshold. The data is publicly available at the following https://doi.org/10.26037/yareta:md2ryexqsrchhb2fafgor6lcmm.

## Results

### Participants

779 physicians were contacted via email (or physical mail where email addresses were unavailable), and followed up by a second email and a telephone call. The response rate was 24% for this first stage. 133 eligible physicians went on to complete both stages of the study (81.1% from the first stage). Difficulty recruiting less experienced physicians was encountered and so the category was extended by increasing the number of years of practice from < 5 to < 6. This enabled 5 participants who had initially been discarded to complete the second stage when recontacted. Their data are included in the second response rate. The desired number of participants was not achieved in the less-experienced psychiatrist category, with a final total of 13 participants. In the other categories the desired number of 40 participants was reached. Participant characteristics displayed a good range of age, years in practice, and site of practice and an equal gender distribution (Table 1). The 40 participants who only completed the first stage of the experiment were statistically compared to the participants who completed both stages and were found to be similar with regard to gender, experience, age, and specialty.

**Table 1:** Participant characteristics

Age (years)	Mean (SD) Median Range	39 (11) 36 24–72
Gender	Male Female	49% 51%
Years in practice	Mean (SD) Median Range	14 (11) 10 1–47
	Less than 6 More than 8	40% 60%
Speciality	Internal medicine Psychiatry	60% 40%
Site of practice	Hospital Hospital ambulatory Private practice	59% 23% 18%

### Overall implicit and explicit prejudice and level of concern among physicians

Despite incomplete comparability due to different stimuli, data do suggest greater implicit prejudice towards the obese than towards the mentally ill among physicians overall (D-score 0.53 and 0.09, respectively, Table 2).

**Table 2:** Specialty, experience, and gender

	All physicians			Psychiatrists Mean (SD)	Internists Mean (SD)	Effect size (95% CI)	p	Less experienced Mean (SD)	More experienced Mean (SD)	Effect size (95% CI)	p
	Mean (SD)	Effect size (95% CI)	p								
Mental Illness IAT (D score)	0.09 (0.50)			− 0.23 (0.42)	0.31 (0.44)	1.25 (0.87–1.63)	** < 0.001**	0.14 (0.50)	0.06 (0.51)	− 0.158 (− 0.506 to 0.19)	0.398
Weight IAT (D score)	0.53 (0.36)			0.50 (0.39)	0.54 (0.34)	0.11 (− 0.236 to 0.458)	0.674	0.56 (0.33)	0.50 (0.38)	− 0.166 (− 0.514 to 0.181	0.392
Feeling Thermometer Mentally Ill (Score 0–12)*	3.96 (2.28)	0.409 (0.065–0.752)	** < 0.001**	2.69 (1.85)	4.80 (2.16)	1.033 (0.813–0.109)	** < 0.001**	4.58 (2.21)	3.55 (2.25)	− 0.461 (− 0.813 to 0.109)	**0.012**
Feeling Thermometer Obese (Score 0–12)*	4.89 (2.27)			5.11 (2.69)	4.75 (1.95)	− 0.158 (− 0.506 to 0.189)	0.374	4.85 (1.94)	4.92 (2.48)	0.031 (− 0.316 to 0.378)	0.876
Vignette overall (Total score 6–42)#	29.94 (3.34)			30.15 (3.17)	29.80 (3.47)	− 0.104 (− 0.452 to 0.243)	0.556	29.08 (3.33)	30.51 (3.24)	0.437 (0.085- 0.788)	**0.014**
Vignette/Depressive patient	29.98 (3.58)	− 0.021 (− 0.361 to 0.319)	0.914	30.86 (2.93)	29.44 (3.86)	− 0.403 (− 0.754 to 0.053)	0.144	28.47 (2.93)	30.61 (3.66)	0.617 (0.041–1.194)	**0.038**
Vignette/Non-depressive patient	29.91 (3.18)			29.65 (3.28)	30.09 (3.13)	0.138 (− 0.23 to 0.485)	0.544	29.36 (3.51)	30.41 (2.78)	0.34 (− 0.01 to 0.689)	0.154
Vignette/Obese patient	29.32 (3.34)	0.39 (0.047–0.733)	0.058	29.15 (2.86)	29.43 (3.59)	0.084 (− 0.263 to 0.432)	0.734	28.97 (3.19)	29.62 (3.41)	0.196 (− 0.152 to 0.543)	0.418
Vignette/Non-obese patient	30.61 (3.28)			31.19 (3.18)	30.21 (3.33)	− 0.3 (− 0.649 to 0.049)	0.244	29.24 (3.61)	31.28 (2.93)	0.65 (0.072–1.227)	**0.018**

P values in bold are statistically significant

The overall mean score for physicians would not count as a mental illness prejudice according to Project Implicit scoring conventions, while obesity prejudice would count as moderate [42, 43]. When a one value t-test was performed to compare the bias to 0, the p value for the Mental Illness IAT was 0.033 and for the Weight IAT was < 0.001.

Explicit feelings were significantly warmer towards the mentally ill than towards the obese (3.94 and 4.89, respectively, p < 0.001, Table 2).

Cronbach’s alpha for the responses to the vignette was 0.79 thus we examined responses together rather than individually, as ‘level of concern for the fictional patient’. There were no significant effects found.

No significant gender effects were found in the results.

### Effect of specialty on prejudice

Compared to internists, psychiatrists appeared to show significantly less implicit bias against mentally ill vs physically ill people (D-score − 0.23 and 0.31, respectively, p < 0.001). The overall mean score for psychiatrists showed a slight positive bias and the overall mean score for internists showed a slight negative bias according to Project Implicit scoring conventions [42, 43]. No significant effects were found for specialty on the Weight IAT (Table 2).

Psychiatrists displayed significantly warmer explicit feelings towards the mentally ill on the Feeling Thermometer than did internists (p < 0.001, Table 2).

### Effect of experience on prejudice

Data did not show significant differences between more or less experienced physicians in either IAT (Table 2).

More experienced physicians showed significantly warmer feelings towards the mentally ill (p = 0.012, Table 2).

The level of concern for the fictional patient was significantly greater among more experienced physicians (p = 0.014, Table 2). Experience was significantly correlated with an increased level of concern when the fictional patient was described as depressed and when he was not described as obese (p = 0.038, p = 0.018, Table 2). When the fictional patient was obese, experience did not correlate with an increased level of concern.

### Effect of interventions on prejudice

Data showed no significant effects of the interventions or of the cognitive load on the IAT or Feeling Thermometer results (Table 3).

**Table 3:** Effects of interventions

	Control (N = 34)	Mental illness video (N = 33)			Obesity video (N = 33)			Cognitive load (N = 33)		
	Mean	Mean (SD)	Effect size (95% CI)	p	Mean (SD)	Effect size (95% CI)	p	Mean (SD)	Effect size (95% CI)	p
Mental illness IAT (D score)	0.043 (0.44)	0.031 (0.37)	− 0.029 (− 0.508 to 0.45)	0.898	0.012 (0.47)	− 0.068 (− 0.547 to 0.411)	.860	0.005 (0.38)	− 0.092 (− 0.572 to 0.387)	0.778
Weight IAT (D score)	0.382 (0.30)	0.386 (0.38)	0.012 (− 0.467 to 0.491)	0.466	0.372 (0.44)	− 0.027 (− 0.506 to 0.452)	.632	0.240 (0.30)	0.352 (− 0.131 to 0.834)	0.058
Feeling thermometer mentally Ill (Score 0–12)*	3.944 (2.10)	3.873 (2.69)	− 0.029 (− 0.508 to 0.45)	0.802	3.967 (2.22)	0.011 (− 0.468 to 0.49)	.986	4.058 (2.18)	0.053 (− 0.426 to 0.532)	0.780
Feeling thermometer obese (Score 0–12)*	4.903 (1.98)	4.958 (2.32)	0.026 (− 0.454 to 0.505)	0.848	4.321 (2.53)	− 0.257 (− 0.738 to 0.224)	.098	5.382 (2.22)	0.228 (− 0.253 to 0.798)	0.160

P values in bold are statistically significant

^*^Lower scores indicate warmer feelings

^#^All interventions were compared to the control

## Discussion

The findings indicate that specialty correlates with both implicit and explicit mental illness prejudice, but not with obesity prejudice. Although experience did not correlate with implicit bias, it was associated with warmer explicit feelings towards the mentally ill and greater concern for the mentally ill -but not the obese- patient on a clinical vignette.

The physicians overall displayed what Project Implicit qualifies as a moderate implicit bias against the obese [42, 43], slightly weaker than the strong negative bias found among physicians and the general public in a previous study [36]. Previously existing evidence on bias against the mentally ill is less conclusive, partly because different IATs have been used [24, 37, 38]. Our Mental Illness IAT results consisting in a slight positive bias for psychiatrists and a slight negative bias for internists indicate that internists may have similar levels to the general population, whereas psychiatrists display less [39]. However, caution should be taken with the interpretation of these results given that the comparison category used in our IAT was physical illness. Another explanation of the difference between psychiatrists and internists is that, while they had similar levels of implicit prejudice towards the mentally ill, internists had a more positive bias towards physical illness than the psychiatrists.

Scores appear to show a greater implicit obesity prejudice than implicit mental illness prejudice, but image-IATs produce different effects from word-IATs due to the level of stimulus representation so cannot be directly compared [56]. However, this difference was reproduced on other measures: greater obesity prejudice than mental illness prejudice was found in the responses to the vignette and on the Feeling Thermometer. The lack of mental illness prejudice on the vignette could be due to our choice of mental disorder. Depression is not associated with such negative implicit stereotypes as other mental disorders, such as schizophrenia [47]. There may be more awareness among physicians of a risk of bias against the mentally ill than against the obese because of the wider prevalence of campaigns against mental illness stigma.

Compared to internists, psychiatrists showed significantly less implicit bias against mentally ill vs physically ill people. The lack of a significant correlation with experience suggests that it may be a self-selection effect (which could take place either before or soon after entry into specialization) rather than the result of training or experience. Perhaps those who choose to specialise in psychiatry already have less implicit bias against the mentally ill or have their initial levels of implicit bias lowered very soon after entering the speciality. If this is the case, this finding is of particular practical importance to the specialty of psychiatry. Alternative hypotheses could include that physicians have more positive associations with diseases they feel better equipped to treat. Greater familiarity, or salience, has also been proposed as a confounding factor in IAT testing [57] and could be at play here.

In contrast to other studies suggesting that training affects implicit prejudice, we found no association between experience and implicit bias [24]. However, the more experienced physicians displayed warmer explicit feelings towards the mentally ill and a higher level of concern for the fictional patient in the vignette. This could indicate that experience in the field improves patient care by making physicians more sympathetic to patients, in addition to other ways. This is particularly interesting in light of other studies suggesting that clinical exposure may erase empathy in medical students [33, 34]. When the fictional patient was described as obese, experience did not correlate with an increased level of concern. One explanation could be that experience helps physicians to prevent their implicit attitudes influencing their level of concern for patients, rather than reducing their implicit mental illness prejudice. This may not occur in the case of obese patients because of lack of awareness of the dangers of obesity prejudice.

Unlike other studies, no gender effects were found in our study. The interventions we tested had no significant effects and the cognitive load procedure made no difference to the IAT scores.

A limitation of the study is that it may include a higher percentage of physicians interested in improving patient care than average because those who participated may have been motivated by this, making results vulnerable to a self-selection effect. Another limitation is the small difference in our experienced and less experienced categories: experienced = practicing more than 8 years; less experienced = (after adjustment) practising for less than 6 years, leaving a possible difference of just over 2 years. A separate analysis was run to compare actual number of years of practice, but found the same correlations. Failure to recruit less experienced psychiatrists also limited possible analyses of differences between experienced and less experienced physicians. In one study, it appears that using different scoring algorithms for the IAT may affect the direction in which cognitive load moderates the scores on the IAT [58] and it is a limitation of our study that we did not investigate this. On the other hand, there was a striking lack of an effect in any direction of the cognitive load on the IAT results.

## Conclusions

Specializing in psychiatry correlates with lower implicit and explicit mental illness prejudice compared to specializing in general medicine. Experience does not correlate with implicit prejudice, but it does correlate with warmer explicit feelings towards the mentally ill and greater concern towards a fictional patient provided they are not described as obese.

In terms of implications for healthcare practice, further research is needed to tease out potentially interacting factors and thus to help the medical profession tailor implicit and explicit bias interventions to specific groups. Psychiatrists may already be less implicitly biased towards the mentally ill when they enter specialization, in which case they will need a different approach to mental health bias training from physicians in other specialties.

What we can recommend now based on our results is that medical education and training should be specifically targeted towards the levels and varieties of implicit bias known to exist in specialities. For instance, medical trainees and experienced physicians may require different forms of explicit and implicit bias training.

Our study points to the strength and potential lack of awareness of obesity prejudice, both implicit and explicit, among physicians. The profession thus needs to raise awareness of the seriousness of this damaging prejudice and its consequences for patients and to consider how to tackle it. Quite apart from the harm and injustice it can engender, it is likely to be highly counter-productive for patient interaction. While efforts have been made recently to develop tailored interventions to combat specific racial implicit stereotypes [59, 60], there has been less research into interventions targeting obesity prejudice. One study found that explicit obesity prejudice increased in students over the four years of medical school [61], suggesting that urgent intervention is required.

## Supplementary Information

**Additional file 1**. Clinical vignette.

## Data Availability

The datasets generated and analysed during the current study are available in the Yareta repository: https://doi.org/10.26037/yareta:md2ryexqsrchhb2fafgor6lcmm
