# Supplementary material for: How is physicians’ implicit prejudice against the obese and mentally ill moderated by specialty and experience?
Source: BMC Med Ethics. 2022 Aug 24;23:86. doi: 10.1186/s12910-022-00815-7 (PMC9400557; doi:10.1186/s12910-022-00815-7)
Supplement: Supplementary file 1 — Additional file 1. Clinical vignette. [file 12910_2022_815_MOESM1_ESM.docx]

**How is physicians’ implicit prejudice against the obese and mentally ill moderated by specialty and experience?**

Chloë FitzGerald^1^*, Christian Mumenthaler^2^, Delphine Berner^1^, Mélinée Schindler^1,^ Tobias Brosch^3^, Samia Hurst^1*^

^1 iEH2 (Institute for Ethics, History and the Humanities), Faculty of Medicine, University of Geneva, Switzerland^

^2 Department of Information Science, Geneva School of Business Administration, University of Applied Sciences and Arts Western Switzerland, Switzerland^

^3 CISA (Swiss National Centre for Affective Sciences), University of Geneva, Switzerland; Department of Psychology, University of Geneva, Switzerland^

^* Corresponding authors: cnsfitzgerald@gmail.com (CF) samia.hurst@unige.ch (SH)^

# Appendix: Clinical Vignette

*N.B. The vignette and questions were in French and have been translated to English.*

Vignette 1: neither obese nor depressed

You have a maximum of **2 minutes** to carefully read the information below relating to this clinical case. This will allow you to form a personal impression of the case on which you will then be asked some questions. Once you have turned the page, you will not be able to consult this written information again to answer the questions that follow.

A 37-year-old man with no particular history arrives in the ER for lower back pain that has spread to his right leg. During this first interview, he is calm and quiet, speaking of his pain as follows:

*"I've been suffering from lower back pain for the past three years and it has recently worsened dramatically. My back and right leg hurt so much that I sometimes have difficulty walking. And even sleeping. It is a very sharp and penetrating pain."*

Vignette 2: obese

You have a maximum of **2 minutes** to carefully read the information below relating to this clinical case. This will allow you to form a personal impression of the case on which you will then be asked some questions. Once you have turned the page, you will not be able to consult this written information again to answer the questions that follow.

A 37-year-old man with a BMI of 32 and no other history arrives in the ER for lower back pain that has spread to his right leg. During this first interview, he is calm and quiet, speaking of his pain as follows:

*"I've been suffering from lower back pain for the past three years and it has recently worsened dramatically. My back and right leg hurt so much that I sometimes have difficulty walking. And even sleeping. It’s a very sharp and penetrating pain."*

Vignette 3: depression

You have a maximum of **2 minutes** to carefully read the information below relating to this clinical case. This will allow you to form a personal impression of the case on which you will then be asked some questions. Once you have turned the page, you will not be able to consult this written information again to answer the questions that follow.

A 37-year-old man, known for several major episodes of depression and with no other history, arrives in the ER for lower back pain that has spread to his right leg. During this first interview, he is calm and quiet, speaking of his pain as follows:

*"I've been suffering from lower back pain for the past three years and it has recently worsened dramatically. My back and right leg hurt so much that I sometimes have difficulty walking. And even sleeping. It’s a very sharp and penetrating pain."*

Vignette 4: obese and depression

You have a maximum of **2 minutes** to carefully read the information below relating to this clinical case. This will allow you to form a personal impression of the case on which you will then be asked some questions. Once you have turned the page, you will not be able to consult this written information again to answer the questions that follow.

A 37-year-old man, known for several major episodes of depression and a BMI of 32 but with no other history, arrives in the ER for lower back pain that has spread to his right leg. During this first interview, he is calm and quiet, speaking of his pain as follows:

*"I've been suffering from lower back pain for the past three years and it has recently worsened dramatically. My back and right leg hurt so much that I sometimes have difficulty walking. And even sleeping. It’s a very sharp and penetrating pain."*

Questions

For each of the following questions, please circle the number that best corresponds to your personal opinion:

1. How do you rate the intensity of the pain presented by the patient?

| 1 | 2 | 3 | 4 | 5 | 6 | 7 |
| --- | --- | --- | --- | --- | --- | --- |
| Not intense at all | Not very intense | Slightly intense | Moderately intense | Intense | Very intense | Extremely intense |

1. How do you assess the severity of the impact of the pain on this man's daily life?

| 1 | 2 | 3 | 4 | 5 | 6 | 7 |
| --- | --- | --- | --- | --- | --- | --- |
| Not severe at all | Not very severe | Slightly severe | Moderately severe | Severe | Very severe | Extremely severe |

1. To what extent do you consider the clinical situation to be of concern?

| 1 | 2 | 3 | 4 | 5 | 6 | 7 |
| --- | --- | --- | --- | --- | --- | --- |
| Not concerning at all | Not very concerning | Slightly concerning | Moderately concerning | Concerning | Very concerning | Extremely concerning |

1. How do you rate the urgency of medical assistance for this patient?

| 1 | 2 | 3 | 4 | 5 | 6 | 7 |
| --- | --- | --- | --- | --- | --- | --- |
| Not urgent at all | Not very urgent | Slightly urgent | Moderately urgent | Urgent | Very urgent | Extremely urgent |

1. How urgent is it for you to relieve this patient's pain?

| 1 | 2 | 3 | 4 | 5 | 6 | 7 |
| --- | --- | --- | --- | --- | --- | --- |
| Not urgent at all | Not very urgent | Slightly urgent | Moderately urgent | Urgent | Very urgent | Extremely urgent |

1. How credible do you think this patient's pain is?

| 1 | 2 | 3 | 4 | 5 | 6 | 7 |
| --- | --- | --- | --- | --- | --- | --- |
| Not credible at all | Not very credible | Slightly credible | Moderately credible | Credible | Very credible | Perfectly credible |
